# Removal of Enantiomeric Ibuprofen in a Nanofiltration Membrane Process

**DOI:** 10.3390/membranes10120383

**Published:** 2020-11-30

**Authors:** Carlyn J. Higgins, Steven J. Duranceau

**Affiliations:** 1 Hazen and Sawyer, 1000 N. Ashley Dr. Suite 1000, Tampa, FL 33602, USA; chiggins@hazenandsawyer.com; 2 Department of Civil, Environmental and Construction Engineering, University of Central Florida, 4000 Central Florida Blvd., Orlando, FL 32816, USA

**Keywords:** nanofiltration, ibuprofen, adsorption, enantiomer, chirality, removal

## Abstract

A study of the behavior of R- and S-enantiomers of ibuprofen (R-IBU and S-IBU) in aqueous solution by nanofiltration (NF) membranes revealed that up to 23% of the pharmaceutical was adsorbed onto the stainless steel equipment of a flat-sheet experimental unit. Mass balances disclosed that IBU’s S-enantiomer was primarily responsible for the adsorption onto the equipment. Additional IBU adsorption was also experienced on the NF membrane coupons, verified by increased contact angle measurements on the surfaces. The IBU-equipment adsorptive relationship with and without the membrane coupon were best described by Freundlich and Langmuir isotherms, respectively. At a feed water pH of 4.0 units and racemic µg/L IBU concentrations, NF removal ranged from 34.5% to 49.5%. The rejection of S-IBU was consistently greater than the R-enantiomer. Adsorption onto the surfaces influenced NF rejection by 18.9% to 27.3%. The removal of IBU displayed a direct relationship with an increase in feed water pH. Conversely, the adsorption of IBU exhibited an indirect relationship with an increase in feed water pH.

## 1. Introduction

The existence and subsequent discovery of chemicals of emerging concern (CECs) in aquatic environments has sparked interest in determining removal capabilities of specific water treatment technologies. Nanofiltration (NF) is a promising pressure-driven semipermeable membrane technology that can be employed to remove CECs from aqueous streams [1,2,3,4,5]. In a membrane process, the extent of solute removal is dependent on chemical properties, feed water matrix composition, membrane characteristics, and operational variables [1,2,6,7,8]. A strong research effort has attempted to elucidate the impact of CEC properties on solute removal through NF processes. It is widely accepted that molecular weight is an important parameter in the prediction of non-charged and non-polar compound rejection [1,9,10,11]. However, other solute characteristics such as chemical properties, solute geometry, and functional groups can also affect rejection of CECs [1,6,7]. In a membrane process, correlations between CEC removal and hydrophobicity [12], membrane adsorption [13], polarizability [14], polarity [15,16], and molecular size and shape [6,7,14,17,18] have been noted. The complexities of CEC mass transfer have been scrutinized and reported on over the years and serve as the basis for additional investigations such as those presented herein.

The position of functional groups in structural isomers has also been shown to have significant effects on rejection by reverse osmosis membranes [19]. This suggests that the spatial arrangement of atoms plays a larger role in membrane process removal than currently understood. A solute property that has received little attention regarding behavior in a membrane process is chirality. Chiral molecules, or stereoisomers, are molecules with the same molecular formula and chemical bonding arrangement, but dissimilar spatial position of atoms. Enantiomers are pairs of stereoisomers that are non-superimposable mirror images. Although enantiomers have the same molecular formula and other chemical properties, some are known to behave differently. 

A well-known example of a chiral molecule is ibuprofen (IBU). IBU is a weak propionic acid derivative and pharmaceutically active compound (PhAC) known for its non-steroidal anti-inflammatory (NSAID) properties. The molecule contains a chiral carbon, yielding two enantiomers, S-IBU and R-IBU. Although medically administered IBU is a racemic mixture of the two enantiomers, the S-form possesses most of the anti-inflammatory properties [20,21,22]. Physiochemical properties of IBU such as low Henry’s law constant (1.5 × 10^−7^ atm-m^3^/mol), moderately high log octanol-water partition constant (K_ow_; 3.97), and soil adsorption constant (log K_oc_; 2.60) suggest that the PhAC often persists in aquatic environments and can display adsorptive qualities to clay and other loamy solids [23]. Like other CECs, IBU has been detected in groundwater and surface waters from the ng/L to µg/L level [24,25]. The enantiomeric ratio (ER) of IBU in surface water has been recorded higher than 0.5, yielding disproportionate enantiomer concentrations in favor of the S-counterpart [26].

In this presented work, the capability of NF process to remove enantiomeric IBU (S-IBU, R-IBU) at acidic pH conditions is explored. The behavior of chirality with respect to removal by nanofiltration membranes has not been fully vetted. The results of this research provide insight into the differing behavior of chiral molecules, further elucidating the effect of solute properties on membrane rejection.

## 2. Materials and Methods 

In this study, Dupont Filmtec NF270 (Edina, MN, USA) and Microdyn Nadir Trisep TS40 (Goleta, CA, USA) membranes were assessed. The polyamide thin-film composite NF270 and polypiperazine amide TS40 were acquired from Sterlitech Corp. (Kent, WA, USA). Membranes were received as flat sheets, cut to the appropriate size, and soaked in deionized water (DI) for at least 24 h prior to experimentation. Membrane operational parameters are listed in Table 1. Racemic IBU was purchased from Sigma-Aldrich (St. Louis, MO, USA) with reported purity of greater than 99%. A 400 mg/L standard solution was prepared in LC/MS grade methanol and sonicated for homogeneity. The standard was stored at 4 °C in a salinized amber bottle and used within one month.

### 2.1. Experimental Setup

A bench-scale, flat-sheet membrane testing apparatus was used in this research. The unit consisted of a Wanner Engineering M-03S Hydra-Cell 6.81 L/min pump (Minneapolis, MN, USA) with a Control Techniques variable frequency drive (VFD) (Eden Prairie, MN, USA), a 19 L Sterlitech stainless steel conical feed tank, two Sterlitech CF042 acrylic cells to house the membrane coupons (operated in parallel for duplicity) with 42 cm^2^ effective membrane area, and accompanying appurtenances consisting of flowmeters, pressure gauges, check valves, and stainless steel braided hose. Two MyWeigh CTS-600 scales (Phoenix, AZ, USA) were utilized for permeate collection and flux measurements. Feed flow was controlled with the VFD and set at 1.0 L/min, corresponding to a crossflow velocity of 0.18 m/s. Feed pressure was controlled by the concentrate control valve. A chiller-coil system was utilized to sustain a feed water temperature of 20 ± 1 °C. 

Prior to each experiment, membrane coupons were inserted into the bench-scale, flat-sheet unit and compacted at 6.9 bar (100 psi) with DI water for at least 24 h. After initial compaction, the mixture was replaced with a 10 L solution containing DI water spiked with racemic IBU and adjusted to acidic conditions (feed water pH of 4.0 to 6.0 units) with 1 M sodium hydroxide or 5.8 M hydrochloric acid. Then, the bench-scale, flat-sheet unit was repressured with the experimental feed water matrix and operated for 24 h. Experiments were conducted in recycle mode, where permeate and concentrate streams recycled back to the feed reservoir. Feed water samples were taken at 0 and 24 h, where permeate and concentrate aliquots were taken at 24 h. The unit was flushed at least twice with 5 L of DI water in between experiments. A new set of membrane coupons was used for each experiment. 

### 2.2. Analytical Methods

Samples were collected in 150 mL salinized amber bottles, stored in a 4 °C refrigerator, and extracted and analyzed within 48 h and 7 d, respectively. A solid phase extraction (SPE) method was utilized to extract and preconcentrate R- and S-IBU enantiomers [27]. Extractions were performed utilizing a Waters vacuum manifold and Waters Oasis HLB 3 mL, 60 mg cartridges (Milford, MA, USA). The R- and S-IBU enantiomers were analyzed via a Perkin-Elmer Series 200 high performance liquid chromatography (HPLC) instrument (Santa Clara, CA, USA). Separations were carried out on a Chiral Technologies, Inc., (West Chester, PA, USA) Chiralcel OJ-H column (4.6 × 150 mm, i.d., 5 µm particle size). The column was operated in polar phase mode, with an isocratic mobile phase consisting of methanol/formic acid (100:0.1, *v*/*v*) at a flow rate of 1 mL/min.

A ramé-hart Model 100 goniometer (Succasunna, NJ, USA) was utilized to determine membrane hydrophobicity via contact angle. Contact angle measurements were attained utilizing the sessile drop technique. Membrane coupons were dried and inserted on the stage with the active layer facing up. A micrometer syringe delivered a droplet of DI water onto the membrane surface, and a contact angle was measured by the goniometer. To obtain a representative contact angle of the entire membrane surface, ten contact angle measurements were taken on various areas of the membrane coupon and averaged.

## 3. Results and Discussion

Adequate mass balance tests are recommended in bench-scale membrane filtration experiments to confirm that rejection is not affected by solute behavior such as volatilization, adsorption, or a reaction with the feed water matrix. Consequently, prior to the series of pressurized filtration tests, a mass balance confirmation experiment was conducted by circulating a feed solution containing 100 µg/L IBU at feed water pH of 4.0 units through the flat-sheet equipment without a membrane coupon for 24 h. After analysis, 23% loss of IBU was observed during the experiment. 

### 3.1. Effect of Feed pH on Adsorption 

**Figure 1:**
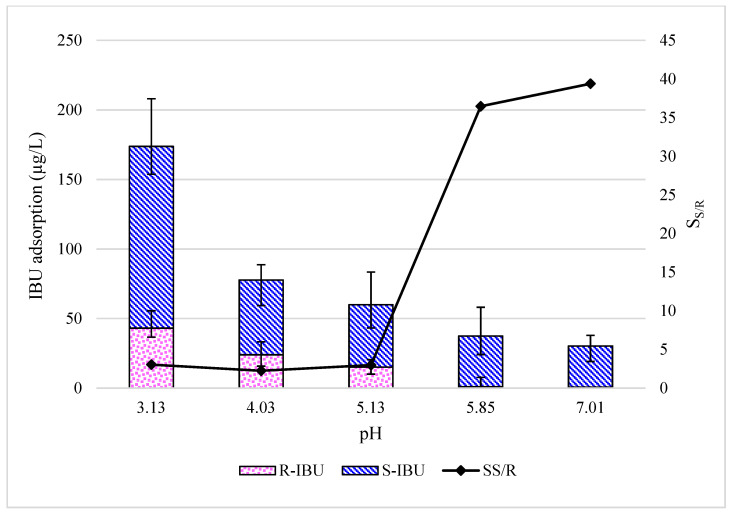
R- and S-enantiomers of ibuprofen (R-IBU and S-IBU) adsorption onto flat-sheet equipment as a function of feed water pH (temperature 20 ± 1 °C). Selectivity is a ratio of adsorption of S/R IBU.

The test was repeated at a feed water pH range from 3.0 to 7.0 units, resulting in an inverse relationship between loss of enantiomeric IBU and the water quality parameter, aligning with the acid dissociation constant (pKa) of IBU (4.4). Figure 1 illustrates the results of the flat-sheet equipment adsorption experiments, where the total IBU concentration adsorbed is presented as a function of the total initial IBU concentration.

Due to the low Henry’s law constant (1.5 × 10^−7^ atm-m^3^/mol), volatilization could not explain observed IBU losses. However, in acidic conditions, IBU is known to adsorb onto and protect metal from corrosion [28,29], and IBU has been documented to attach to chromium-based metal organic frameworks [30]. It is noted that the flat-sheet test equipment is comprised mainly of components that consist of stainless steel (16% chromium, 10% nickel, 2% molybdenum, and less than 0.02% carbon) [31]. Existing literature suggests that IBU adsorption onto the flat-sheet stainless steel components (comprised of the reservoir, tubing, and chiller coil) could occur, as similar results have been realized with 9-anthracenecarboxylic acid [32]. Minimal to no attachment to the acrylic holding cells and permeate polyethylene tubing was observed. The bonding mechanism of IBU adsorption onto the surface was postulated to be between the hydrogen on the carboxylate functional group of the solute and adsorption of oxygen on the metal from the hydroxide moiety [33,34].

### 3.2. Effect of Feed Concentration on Adsorption 

Although significant adsorption onto the flat-sheet equipment was observed, IBU has also been known to attach onto membrane surfaces at pH values less than its pKa of 4.4 [35]. Hence, experiments deciphering the extent of IBU adsorption onto the flat-sheet equipment with and without a membrane coupon installed were conducted by repeating the experiment for a racemic solute concentration range from 100 µg/L to 1.50 mg/L. Feed samples were collected at 0 and 24 h. Triplicate feed concentration measurements were taken and averaged. 

**Figure 2:**
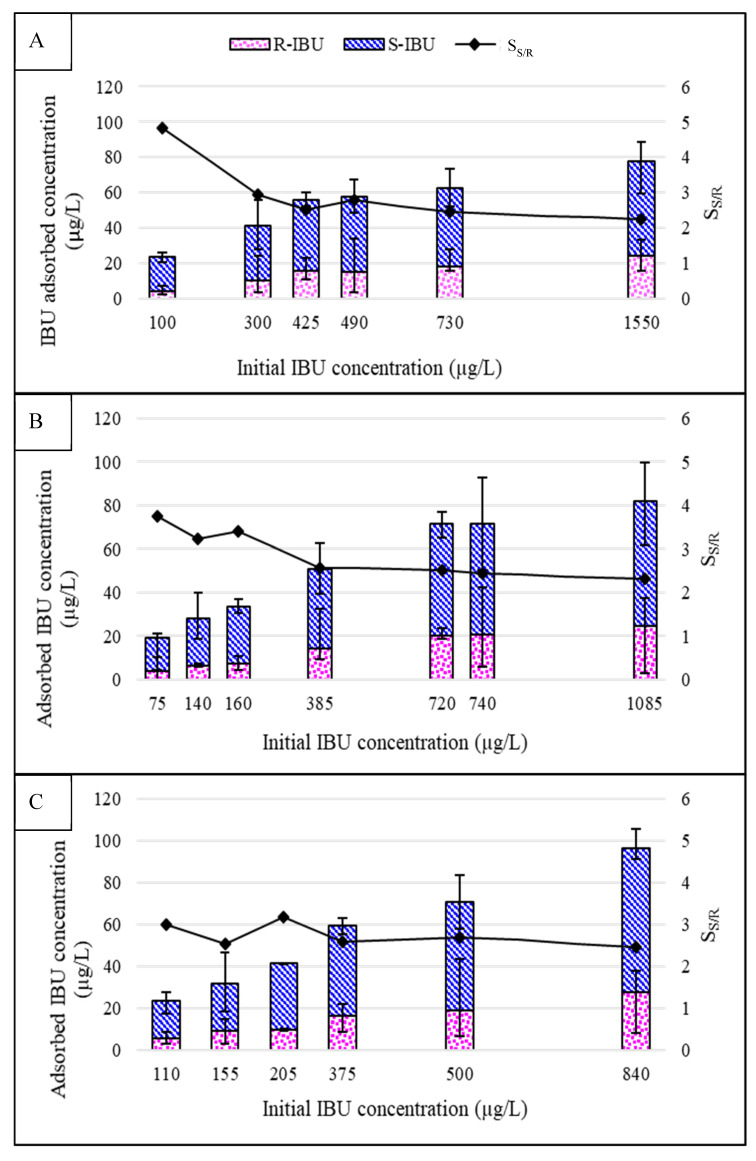
Adsorbed R- and S-IBU onto (**A**) Flat-sheet equipment; (**B**) Flat-sheet equipment with NF270 coupon; (**C**) Flat-sheet equipment with TS40 coupon (feed water pH of 4.0 units, temperature 20 ± 1 °C). Selectivity is a ratio of adsorption of S/R IBU. Error bars represent minimum and maximum values from triplicate analysis.

Results in Figure 2 indicate that the adsorption of IBU increases with initial feed concentration, which agree with prior PhAC-metal attachment studies [29]. It also appears that the sorbed IBU concentration may approach a saturated equilibrium in due course, and hence can be modeled by adsorption isotherms (see Supplementary Information).

**Figure 3:**
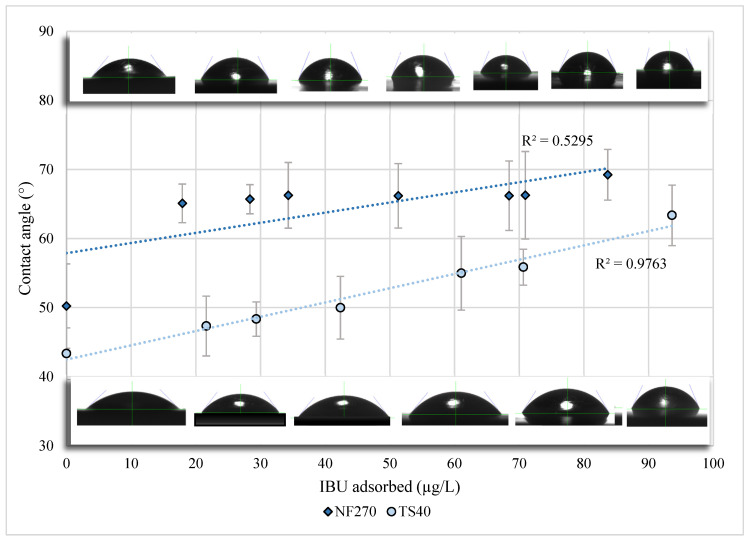
Contact angle of NF270 and TS40 membranes as a function of IBU adsorption. Error bars represent one standard deviation of uncertainty. Contact angle snapshots provide a visual image of the linear relationship between hydrophobicity and adsorbed IBU.

An additional 19.6% to 39.2% IBU adsorption onto flat-sheet equipment with membrane coupon was recorded. These results suggest that additional IBU adsorption presumably occurred onto the membrane surface. IBU adsorption onto the membrane components was validated by an increase in hydrophobicity (measured by contact angle), illustrated in Figure 3. Hydrophobicity was found to have a positive direct relationship with the concentration of adsorbed IBU. Mechanisms of adsorption could include both hydrophobic interactions and the formation of hydrogen bonds between IBU and the membrane surface [36]. Previous studies have attributed IBU adsorption only to the membrane surface, neglecting to fully understand the behavior of IBU, hence, conceivably reporting inaccurate rejection values in flat-sheet studies [37,38,39,40]. 

At an initial racemic concentration of 1.08 mg/L, 83.7 µg/L IBU adsorbed onto the equipment and NF270 membrane. On the contrary, at an initial racemic content of 840 µg/L, 93.6 µg/L IBU adsorbed onto the equipment and TS40 membrane. Therefore, the TS40 membrane contained a slightly higher capacity to adsorb IBU. The difference of adsorption could not be explained by pore size or surface hydrophobicity. Although the NF270 membrane is more hydrophobic and has a larger MWCO, it did not adsorb as much IBU as the TS40 component. Others have postulated similar findings [35]. It should be noted that static batch experiments investigating IBU adsorptive capabilities on membrane coupons have been conducted elsewhere [35]. Batch adsorption experiments often do not represent actual attachment capacities of membrane while in pressurized operation [32], and thus were not included in the scope of this work.

An apparent difference between the adsorption of R- and S-IBU onto the metal surface was noted. At an initial racemic concentration of 100 µg/L, S-IBU adsorbed 4.82 times more than its R-counterpart. The ratio fell to 2.25 at an initial racemic concentration of 1.50 mg/L. Although current literature on the adsorption behavior of enantiomers is scarce, some have claimed IBU can enantioselectively adsorb onto chromium- and vanadium-based metal organic frameworks [30]. Additionally, S-IBU has been reported to adsorb up to 10 times more than the R-enantiomer on a liposome membrane [41]. In liposomes, enantioselectivity was ascribed to hydrogen bonding or hydrophobic interactions between the asymmetric carbons of the chiral molecule and the spherical vesicle. 

A possible explanation for the disparate enantiomer behavior could reside in optimized molecular geometry between R- and S-IBU performed by density functional theory (DFT) computations [42,43]. In conjunction with experimental studies, DFT computations can provide insight to the contrary behavior of chiral molecules, as previously illustrated by D-alanine’s enantioselective adsorptive behavior [44]. The DFT framework in this study utilized the gradient correction non-local correlation functional of Lee, Yang, and Parr (B3LYP) with a basis set of 6-31G*, using the online GAMESS software [45,46]. Table 2 presents a comparison of the DFT-derived total energies and geometric properties of R- and S-IBU. 

Results indicate approximately equal energies of R- and S-IBU, however, the surface area, volume, and dipole moment differ, which align with findings presented elsewhere [47]. The larger surface area, molecular volume, and smaller dipole moment of R-IBU suggest a bulkier, more hindered approach as compared with S-IBU. It should be noted that the ratio of S- to R-IBU dipole moments (2.68) compares well with the adsorption selectivity at initial racemic concentrations greater than 300 µg/L (2.46). Other conceivable explications for the dissonant chiral behavior include the Easson–Stedman three-point “lock and key” hypothesis between the chemical and binding site [48,49] or the slightly unequal opposite optical rotations of the enantiomers [50]. 

### 3.3. Adsorption Isotherm Modeling 

Adsorption isotherms can be used to describe the relationship between the quantity of IBU attached on a solid surface in relation to its surrounding aqueous concentration at a constant temperature and pressure [51]. The concentration of IBU adsorbed to the solid surface at quasi-equilibrium (q_e_) is calculated by Equation (1):

(1) $$q_{e}=\frac{(C_{o}-C_{e})V}{A}$$

where q_e_ is the concentration of IBU on solid surface (µg/cm^2^), C_o_ is the initial concentration of IBU in aqueous solution (µg/L), C_e_ is the equilibrium concentration of IBU in aqueous solution (µg/L), V is the volume of aqueous solution (L), and A is the surface area of solid surface (cm^2^).

In this work, Langmuir, Freundlich, and Temkin isotherms were utilized to model the adsorption behavior of IBU [51,52,53,54,55,56,57]. Manipulations of q_e_ and C_e_ for R- and S-IBU were plotted in accordance with the Langmuir, Freundlich, or Temkin isotherms to determine the best-fit model for the adsorption system. Isotherms were ascertained for error using coefficient of determination (r^2^), relative percent difference (RPD), some of square errors (ERRSQ), and root mean square error (RMSE) [56]. Derived parameters and statistical error are shown in Table 3. The variables K_L_ and q_a_ represent Langmuir adsorption constants, K_F_ and n represents Freundlich adsorption constants, and the and K_T_ and b represent Temkin adsorption constants. 

From Table 3, the Langmuir, Freundlich, and Temkin isotherms yielded r^2^ values > 0.90, authenticating adsorption equilibrium tendencies for experimental data. Favorable adsorption was observed in the Freundlich isotherm as 1/n values were < 1 for R- and S-IBU. Adsorption intensities denoted by constants K_L_, K_F_, and K_T_ were higher for S-IBU. Furthermore, greater Langmuir maximum adsorption capacities (q_a_) were also observed for S-IBU, aligning with the favorable adsorption presented herein. Larger concentrations of adsorbed IBU on the surface (q_e_) were experienced in the equipment-IBU-membrane system, highlighting the additional adsorptive capacity of the membrane surface. 

The Langmuir, Freundlich, and Temkin isotherms yielded similar predictability for IBU equilibrium concentrations up to 350 µg/L but diverged as solute content increased. Error analysis revealed that the Langmuir isotherm best modeled the equipment-IBU relationship. A Langmuir adsorption model fit insinuates an equal quantity of attachment free-energy changes and a monolater coating of IBU on the surface. Similar results have been realized in applications utilizing stainless steel as the adsorbent [57,58,59]. Therefore, the equipment-IBU relationship can be modeled via Langmuir > Temkin > Freundlich. On the contrary, the Freundlich isotherm revealed the closest representation to the range of equipment-IBU-membrane system experimental data based on error analysis. A best-fit Freundlich adsorption isotherm suggests heterogeneous adsorption free-energy changes and a multilayer of IBU chemisorption. These findings align with existing literature denoting Freundlich-type adsorption on a membrane surface due to its laminose structure [35,60]. However, it should be noted that the Langmuir and Freundlich isotherms produced analogous r^2^ values for R-IBU (0.995 for NF270 and 0.996 for TS40), and contained similar error statistics. This suggests that as S-IBU has a stronger attachment affinity, weak interactions between R-IBU and the surface may yield a thinner adsorptive layer. A study of R- and S-IBU adsorption kinetics onto metal and membrane surfaces may elucidate the dissimilar attachment mechanisms. For the purposes of this work, the equipment-S-IBU-membrane system can be modeled via Freundlich > Temkin > Langmuir, whereas the equipment-R-IBU-membrane system can be modeled via Freundlich = Langmuir > Temkin. 

### 3.4. Rejection of Ibuprofen Enantiomers 

**Figure 4:**
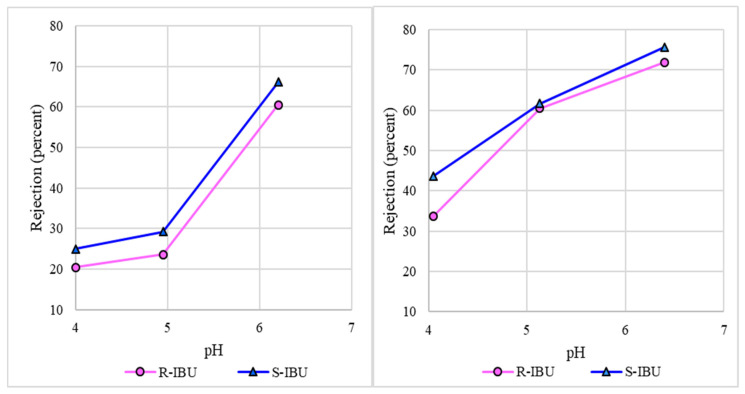
R- and S-IBU rejection as a function of feed water pH for NF270 membrane (**left**), TS40 membrane (**right**) (water flux 42.4 L/m^2^h, temperature 20 ± 1 °C).

The effect of pH on IBU rejection via NF was investigated by altering the feed pH to 4.0, 5.0, or 6.0 units with an initial IBU concentration of 1.5 mg/L (R- and S-enantiomer concentrations of 750 µg/L). Figure 4 displays the rejection of R- and S-IBU from the NF270 and TS40 membrane at a feed pH of 4.0 units. The total NF270 and TS40 IBU rejection was 34.5% and 49%, respectively. However, the adsorption of IBU affected the rejection value based on the time of collection. Adsorption accounted for 14.3% to 23.4% and 23.6% to 31.3% of R-IBU and S-IBU rejection, respectively.

The NF270 and TS40 exhibited poor (<50%) rejection at a feed water pH of 4.0 units. However, removal efficacy increased with feed water pH, as illustrated in Figure 4, aligning with findings from others [37,38]. The feed water pH affects the speciation of IBU and the magnitude of negative charge on the NF membrane. It should be noted that feed water pH has an opposing effect on IBU adsorption. At feed water pH values higher than 4.4 units, the membrane surface is negatively charged and IBU is dissociated, primarily existing in the anionic form. Anionic IBU is believed to be rejected by electrostatic repulsion and steric hindrance. Conversely, at a feed water pH less than 4.4 units, IBU principally exists as the neutral form, and the membrane is less negatively charged. Neutral IBU readily adsorbs onto stainless steel and the membrane surface. As available adsorptive sites become saturated, the NF membrane can partially reject neutral IBU due to size exclusion. Therefore, the mechanism of IBU removal at acidic conditions is postulated as initially adsorption and subsequently steric hindrance. 

**Figure 5:**
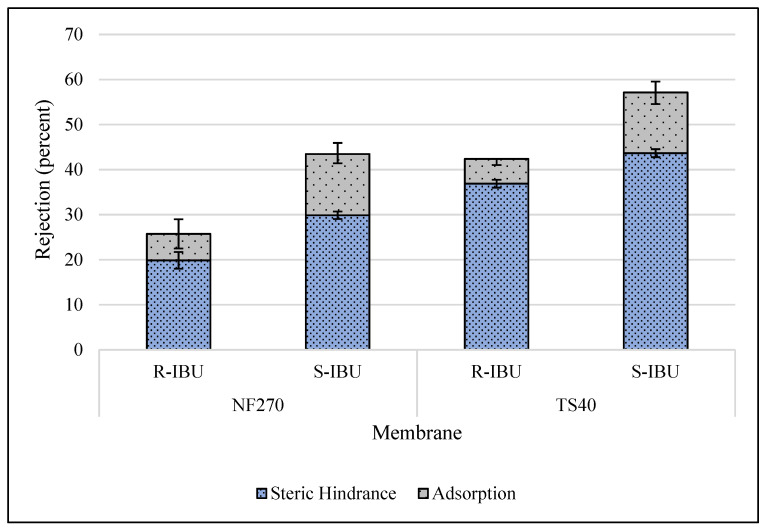
Rejection of R- and S-IBU at a feed water pH of 4.0 units (initial IBU concentration 400 µg/L, water flux 42.4 L/m^2^h, temperature 20 ± 1 °C). Error bars represent minimum and maximum values from triplicate analysis.

It should be noted that the rejection of S-IBU was consistently greater than the R-enantiomer for the feed pH conditions examined, due to the preferential attachment onto the metal flat-sheet equipment, shown in Figure 5. Although CEC adsorption impacts the overall rejection, it should not impact the mechanism of removal by the membrane. This indicates that the membrane may have a slight affinity for the rejection of S-IBU. A possible explanation for the increased rejection lies in DFT calculations, which revealed a dipole moment of 2.02 and 5.40 Debeye for R- and S-IBU, respectively. Existing literature suggests that a molecule’s polarity influences the orientation of the solute relative to the membrane [15,16]. A molecule with a lower dipole moment is less polar, and hence contains an orientation more perpendicular to the membrane surface, increasing the probability of the solute to travel through the material without being rejected. Others have also found a direct relationship between CEC dipole moment and rejection [14,15,16,61].

The influence of sample time is important when recording removal of hydrophobic CECs like IBU from a NF process. In this work, adsorption was recorded over 24 h, and rejection was collected at 24 h. Others have agreed that 24 h of operation was adequate for equilibration of hydrophobic compounds [12,13,32]. However, additional time may be required to confirm complete adsorption of the compound. If rejection is collected shortly after start-up, the value may not account for the adsorption of the CEC onto the membrane or equipment. Therefore, system equilibration is important in obtaining accurate removal capacities.

## 4. Conclusions

The results of this study revealed the behavior of IBU enantiomers in aqueous solutions treated by NF membranes. Feed water characteristics (such as pH) have a substantial influence on the rejection and adsorption mechanisms of IBU. At low feed pH values, S-IBU adsorbed up to five times more than its R-counterpart onto stainless steel and showed preferential rejection in a NF process. DFT calculations could provide insight into the differing behavior of the enantiomers in terms of molecular volume and dipole moment. In bench-scale membrane removal studies, it is important to conduct initial mass balance experiments to determine possible losses of compound, which may impact overall removal. Furthermore, equilibration time proves vital in determination of the true removal capabilities of membrane processes. 

## Figures and Tables

**Table 1:** Membrane operational properties.

Membrane	MWCO (Da)	Water Flux Coefficient (L_p_)	MgSO_4_ Rejection (%)	Contact Angle (Virgin, °)	Contact Angle (Compacted, °)
**NF270**	200–400	0.460	>97	30.6	50.2
**TS40**	200–300	0.231	>98.5	28.7	43.3

**Table 2:** Density functional theory (DFT) calculated energy and geometries of R- and S-IBU.

Compound	Method	Energy (Hartrees)	Surface Area (Å^2^)	Molecular Volume (Å^3^)	Dipole Moment (Debeye)
**R-IBU**	B3LYP/6-31G*	−656.3	179.5	199.3	2.018
**S-IBU**	B3LYP/6-31G*	−656.3	173.1	194.3	5.404

**Table 3:** R- and S-IBU Langmuir, Freundlich, and Temkin isotherm parameters derived from bench-scale, flat-sheet experiments (feed water pH of 4.0 units, temperature of 20 ± 1 °C).

		Isotherm					
		Langmuir					
		K_L_ (L/µg)	q_a_ (µg/cm^2^)	r^2^	RPD	ERRSQ	RMSE
**R-IBU**	Equipment	3.93 × 10^−3^	0.031	0.993	3.73	3.42 × 10^−3^	2.40 × 10^−2^
	NF270	4.08 × 10^−3^	0.033	0.995	4.04	4.98 × 10^−6^	8.43 × 10^−4^
	TS40	3.88 × 10^−3^	0.040	0.996	3.76	6.67 × 10^−6^	1.05 × 10^−3^
**S-IBU**	Equipment	1.74 × 10^−2^	0.052	0.979	3.57	2.46 × 10^−5^	2.02 × 10^−3^
	NF270	1.44 × 10^−2^	0.057	0.982	6.45	6.62 × 10^−5^	3.08 × 10^−3^
	TS40	8.95 × 10^−2^	0.076	0.974	6.00	8.71 × 10^−5^	3.81 × 10^−3^
		**Freundlich**					
		**K_F_ (L/cm^2^)**	**1/n (-)**	**r^2^**	**RPD**	**ERRSQ**	**RMSE**
**R-IBU**	Equipment	6.35 × 10^−4^	0.566	0.945	9.36	3.38 × 10^−3^	2.40 × 10^−2^
	NF270	4.05 × 10^−4^	0.665	0.995	3.87	4.53 × 10^−6^	8.05 × 10^−4^
	TS40	4.74 × 10^−4^	0.677	0.996	2.78	7.99 × 10^−7^	3.65 × 10^−4^
**S-IBU**	Equipment	5.89 × 10^−3^	0.350	0.946	7.97	7.67 × 10^−5^	3.58 × 10^−3^
	NF270	4.00 × 10^−3^	0.433	0.988	3.92	1.80 × 10^−5^	1.60 × 10^−3^
	TS40	2.43 × 10^−3^	0.570	0.994	3.02	1.08 × 10^−5^	1.34 × 10^−3^
		**Temkin**					
		**K_T_ (L/µg)**	**b (J/mol)**	**r^2^**	**RPD**	**ERRSQ**	**RMSE**
**R-IBU**	Equipment	0.040	3.58 × 10^5^	0.981	5.42	3.39 × 10^−3^	2.30 × 10^−2^
	NF270	0.041	3.25 × 10^5^	0.980	14.0	7.47 × 10^−6^	1.03 × 10^−3^
	TS40	0.038	2.65 × 10^5^	0.963	10.4	1.11 × 10^−5^	1.36 × 10^−3^
**S-IBU**	Equipment	0.185	2.23 × 10^5^	0.974	3.56	1.11 × 10^−5^	1.36 × 10^−3^
	NF270	0.110	1.78 × 10^5^	0.988	5.19	1.86 × 10^−5^	1.63 × 10^−3^
	TS40	0.061	1.19 × 10^5^	0.975	9.11	4.23 × 10^−6^	2.66 × 10^−3^

## Supplementary Materials

The following are available online at https://www.mdpi.com/2077-0375/10/12/383/s1, Figure S1: Enantiomers of IBU, Figure S2: IBU speciation based on pKa value (4.4), Figure S3: Flat-sheet bench-scale unit schematic operated in (A) recycle mode, (B), permeate collection mode, Figure S4: HPLC IBU enantiomer chromatogram, Figure S5: Dipole moment of (A) R-IBU and (B) S-IBU produced by DFT computations using GAMESS software. Figures S6–S13: Linearized adsorption isotherms for R- and S-IBU. Figure S14: Adsorption isotherm curves of R- and S-IBU, Figure S15: Adsorption isotherm curves of R- and S-IBU and (A) flat-sheet equipment, (B) flat-sheet equipment and NF270 coupon, (C) flat-sheet equipment and TS40 coupon (feed water pH of 4.0 units, temperature 20 ± 1°C). Error bars represent minimum and maximum values from triplicate analysis, Table S1: Chemical properties of IBU, Table S2: Operational parameters of NF270 and TS40 NF membrane coupons.
