# Supplementary material for: Removal of Enantiomeric Ibuprofen in a Nanofiltration Membrane Process
Source: Membranes (Basel). 2020 Nov 30;10(12):383. doi: 10.3390/membranes10120383 (PMC7761409; doi:10.3390/membranes10120383)
Supplement: Supplementary file 1 [file membranes-10-00383-s001.pdf]

membranes

Supplementary Information: Removal of Enantiomeric Ibuprofen in a Nanofiltration Membrane Process

Carlyn J. Higgins [1] and Steven J. Duranceau [2,*]

[1] Hazen and Sawyer, 1000 N. Ashley Dr. Suite 1000, Tampa FL 33602, USA; chiggins@hazenandsawyer.com
[2] Department of Civil, Environmental and Construction Engineering, University of Central Florida, 4000 Central Florida Blvd., Orlando FL 32816-2450, USA
* Correspondence: steven.duranceau@ucf.edu, Tel: +1-407-823-1440

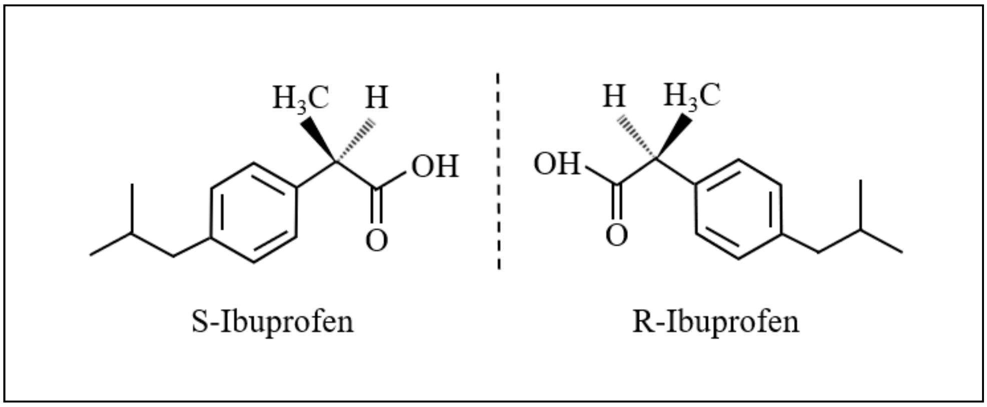

Figure S1. Enantiomers of IBU.

Table S1. Chemical properties of IBU.

Property	Value
Molecular formula	$C_{13}H_{18}O_2$
CAS no.	15687-27-1
Molecular weight (g/mol)[1]	206.28
Density (g/mL)	1.03
Water solubility at 25 °C (g/L)[2]	0.021
Boiling point (°C at 760 mmHg)[1]	157
Melting point (°C)[1]	76
Vapor pressure (mmHg at 25 °C)[4]	4.74×10^{-5}
Octanol-water partition coefficient (log K_{ow})[3]	3.97
Soil organic carbon-water partitioning coefficient (log K_{oc})[6]	2.60
Henry's law constant at 25 °C (atm-m^3/mol)[6]	1.5×10^{-7}
Acid dissociation constant (log K_a)[5]	4.4

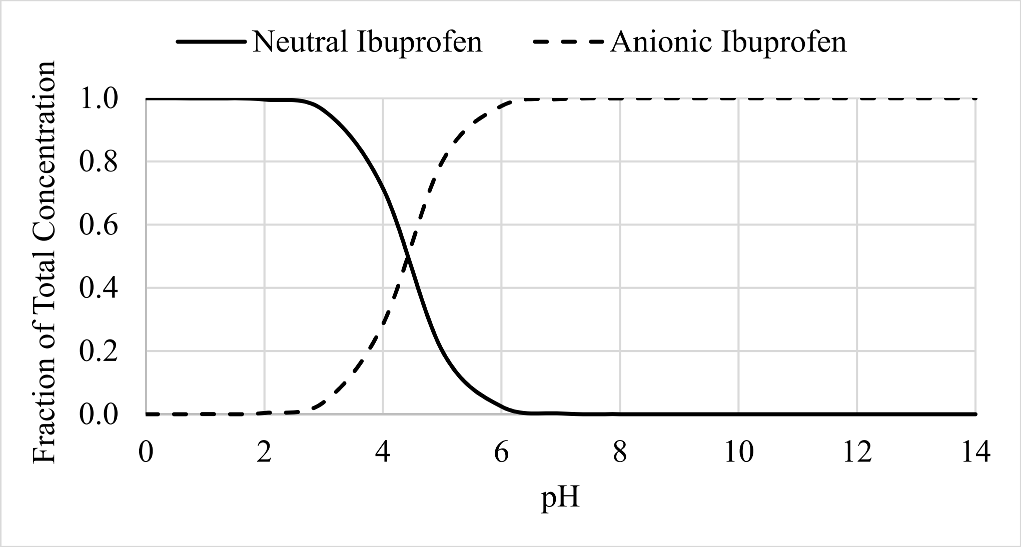

Figure S2. IBU speciation based on pKa value (4.4).

Membrane Properties and Experimental Procedures

Prior to pressurized experiments, membrane operational properties were determined. The water flux coefficient (L_P) was evaluated by collecting permeate water flux at a pressure range from 25 to

200 psi, which satisfies the typical NF pressure range in application. Water flux was determined as a ratio of the permeate flow over the area of the membrane, shown as Equation (S1) [7].

$$J_w = k_w(\Delta P - \Delta \pi) = \frac{Q_P}{A} \quad \text{(S1)}$$

Virgin contact angle was determined upon receipt of the membranes, and compacted contact angle was determined after compaction with DI for 24 h, followed by drying for 48 h. A ramé-hart Model 100 Goniometer (Succasunna, NJ, USA) was utilized to determine membrane hydrophobicity via contact angle. Contact angle measurements were attained utilizing the sessile drop technique [8,9]. Membrane coupons were dried and inserted on the stage with the active layer facing up. A micrometer syringe delivered a droplet of DI water onto the membrane surface, and a contact angle was measured by the goniometer. To obtain a representative contact angle of the entire membrane surface, ten contact angle measurements were taken on various areas of the membrane coupon and averaged.

Table 2. Operational parameters of NF270 and TS40 NF membrane coupons.

Membrane	Membrane Type	MWCO (Da)	Water Flux Coefficient (L_P)	Contact Angle (Virgin, °)	Contact Angle (Compacted, °)
NF270	Polyamide Thin-Film Composite	200–400	0.460	30.6	50.2
TS40	Polypiperazine	200–300	0.231	28.7	43.3

The bench-scale, flat-sheet unit was operated by pumping pressurized feed water through a membrane coupon and producing a permeate and concentrate stream. During each experiment, permeate and concentrate streams were recycled back into the feed reservoir to simulate a constant feed composition. However, permeate tubes were taken from the feed reservoir and collected when appropriate.

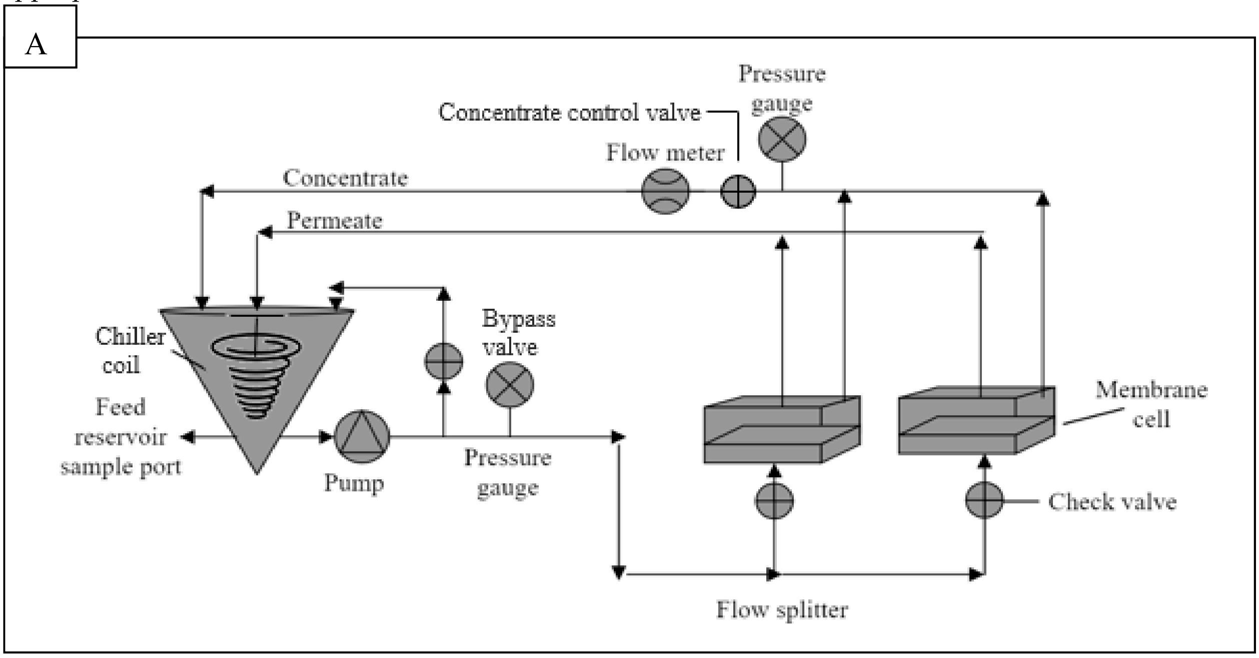

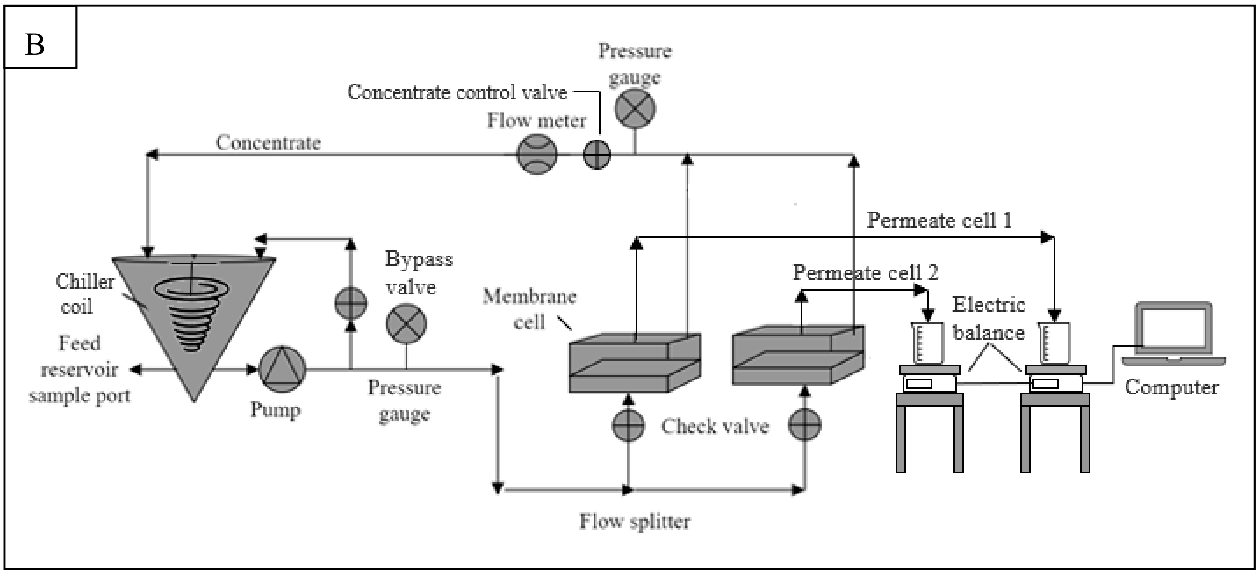

Figure S3. Flat-sheet, bench-scale unit schematic operated in (**A**) recycle mode and (**B**) permeate collection mode.

Solid Phase Extraction Method

A solid phase extraction (SPE) method was utilized to extract and preconcentrate R- and S-IBU enantiomers [10]. Oasis HLB 3 mL, 60 mg cartridges were conditioned by gravity with 3 mL acetonitrile, 3 mL methanol, and then 3 mL HPLC grade water. Then, samples were loaded through the SPE cartridges under vacuum of approximately 4 psi and a constant flow rate of less than 2 mL/min. Then, sample bottles were washed with 6 mL of HPLC grade water, which was also sent through the cartridges. Next, cartridges were dried for 5 min under a vacuum pressure of 10 psi and eluted into sample tubes by gravity with 4 mL acetonitrile. Then, samples were evaporated using an Organomation N-EVAP nitrogen gas evaporator with water bath at 65 °C (Berlin, MA, USA) and then reconstituted with 1 mL methanol/formic acid (100:0.1 v/v) and manually agitated to dissolve the residue.

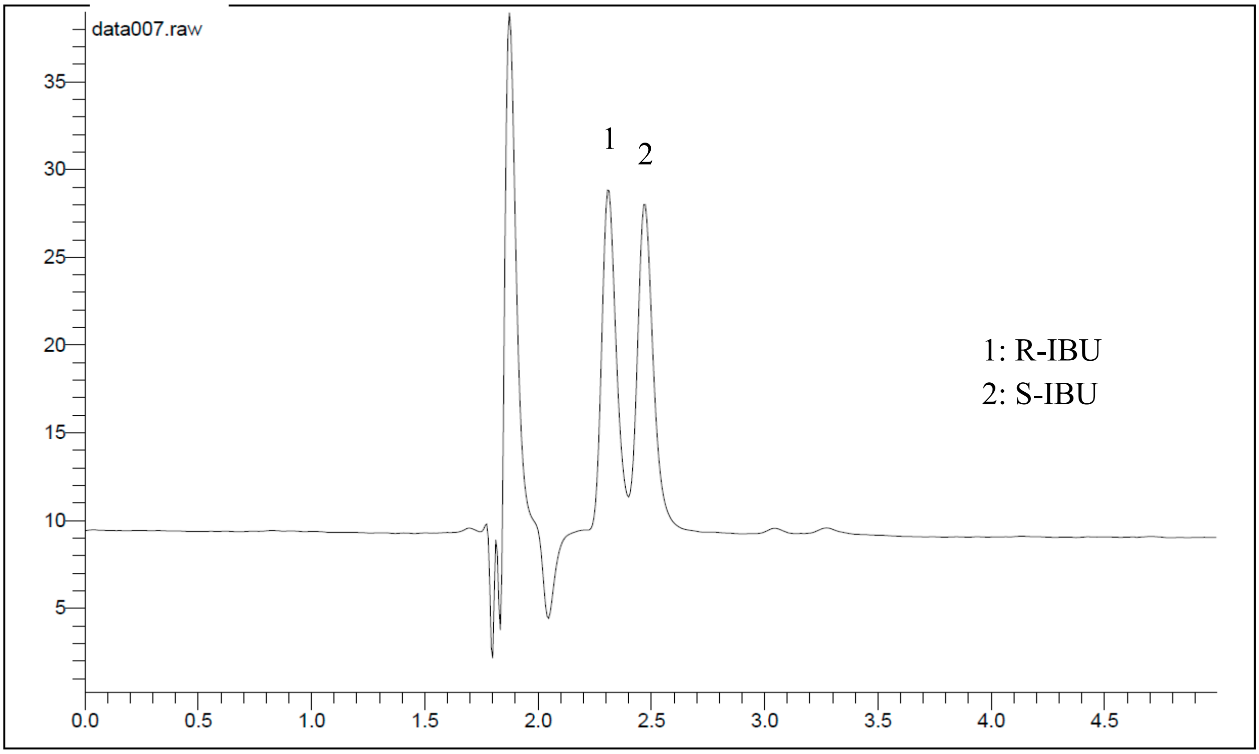

Figure S4. HPLC IBU enantiomer chromatogram.

DFT Games Software Image Output

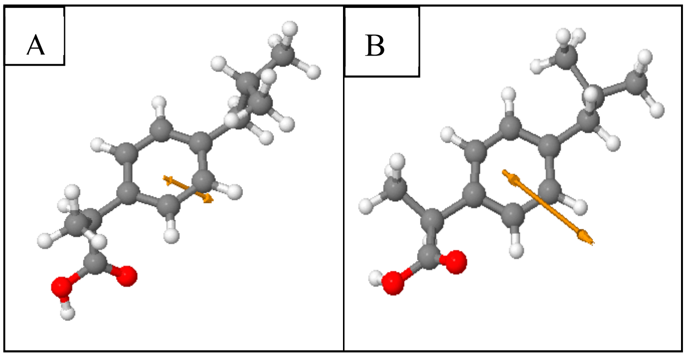

Figure S5. Dipole moment of (**A**) R-IBU and (**B**) S-IBU produced by DFT computations using GAMESS software. Dipole moment is depicted by an orange arrow.

Adsorption Isotherm Modeling

Adsorption isotherms can be used to describe the relationship between the concentration of IBU adsorbed on a solid surface in relation to its surrounding aqueous content at a constant temperature and pressure. In this work, linearized forms of the Langmuir, Freundlich, and Temkin isotherm were used to model the relationship between the equilibrium concentration of IBU adsorbed on a solid surface, shown as Equations (S2) to (S4) [11–13]:

$$\frac{1}{q_e} = \frac{1}{q_a} + \frac{1}{K_L q_a C_e} \tag{S2}$$

$$\log(q_e) = \log(K_F) + (\frac{1}{n}\log(C_e)) \tag{S3}$$

$$q_e = \frac{RT}{b}\ln(K_T) + \frac{RT}{b}\ln(C_e) \tag{S4}$$

where,

q_e = concentration of IBU on solid surface (μg/cm^2)
q_a = maximum adsorption capacity (μg/cm^2)
K_L = Langmuir adsorption constant (L/μg)
C_e = equilibrium concentration of IBU in aqueous solution (μg/L)
K_F = Freundlich adsorption constant (L/cm^2)
$1/n$ = Freundlich adsorption intensity constant, unitless
R = universal gas constant (8.314 J/Kmol)
T = temperature (K)
b = Temkin isotherm constant (J/mol)
K_T = Temkin isotherm equilibrium binding constant (L/μg).

Equipment stainless steel surface area was calculated as 10,247 cm^2, and 10,331 cm^2 with the membrane coupons installed. Adsorption isotherms were considered for equipment-IBU and equipment-IBU-membrane relationships. Slopes and intercepts from the linearized forms of the isotherms were used to calculate appropriate parameters of each adsorption isotherm.

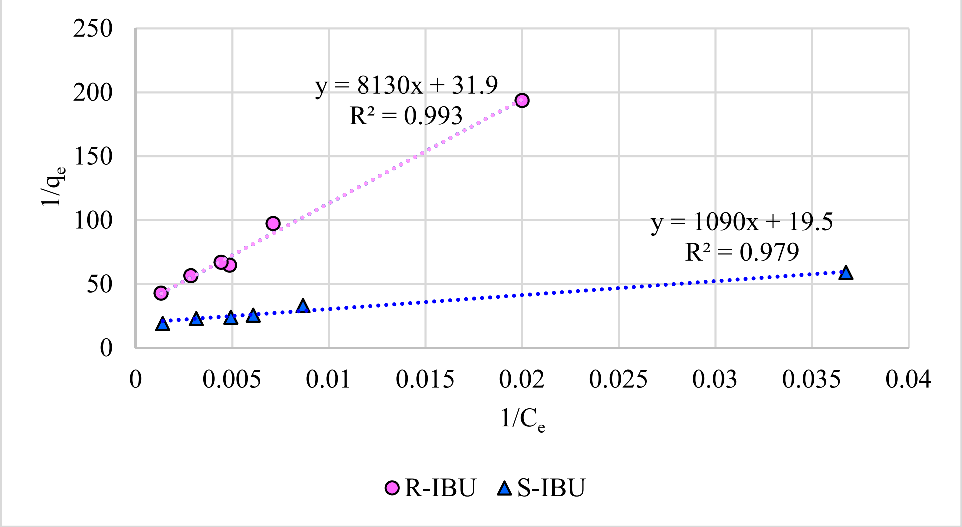

Figure S6. Linearized Langmuir isotherm plot of equipment-IBU adsorption relationship.

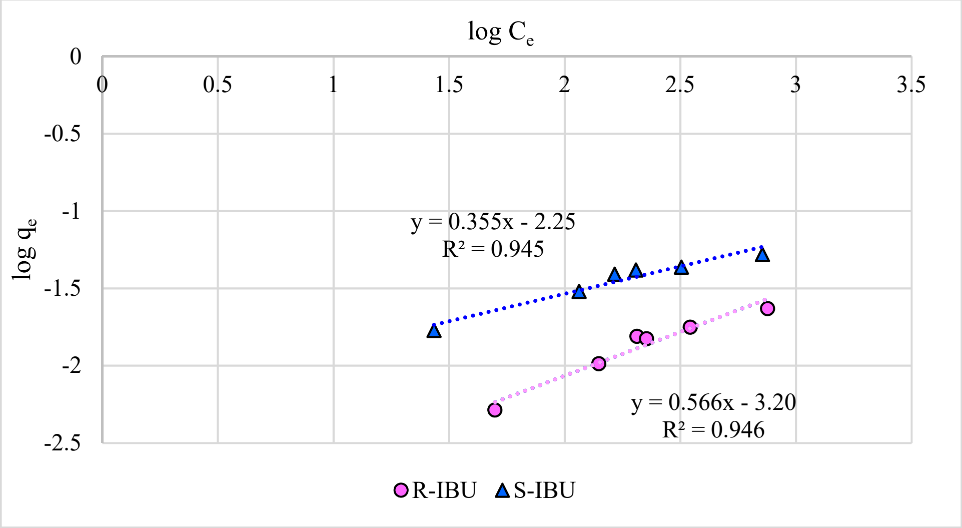

Figure S7. Linearized Freundlich isotherm plot of equipment-IBU adsorption relationship.

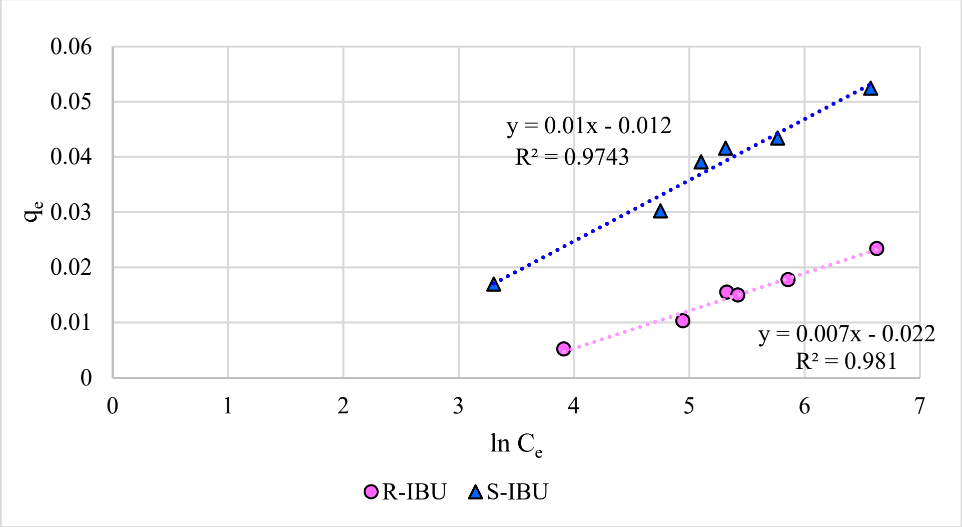

Figure S8. Linearized Temkin isotherm plot of equipment-IBU adsorption relationship.

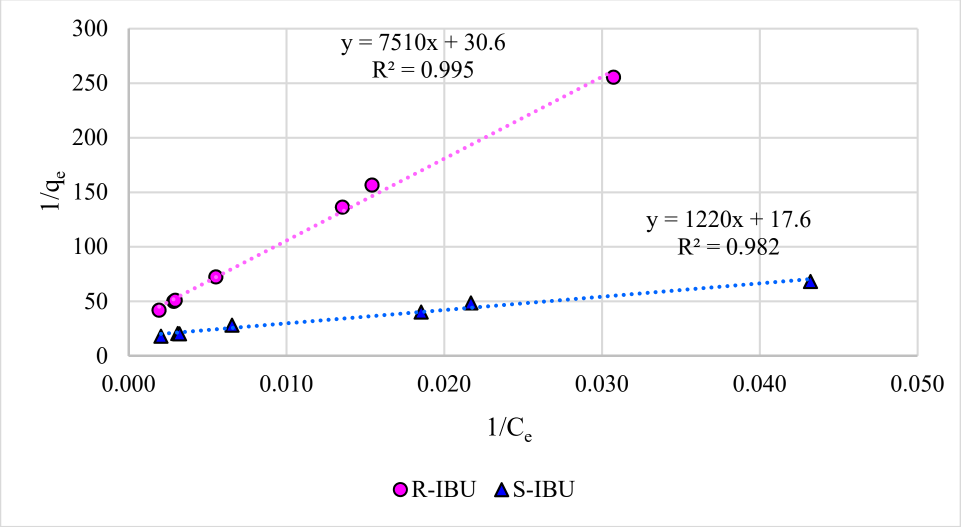

Figure S9. Linearized Langmuir isotherm plot of equipment-IBU-NF270 membrane adsorption relationship.

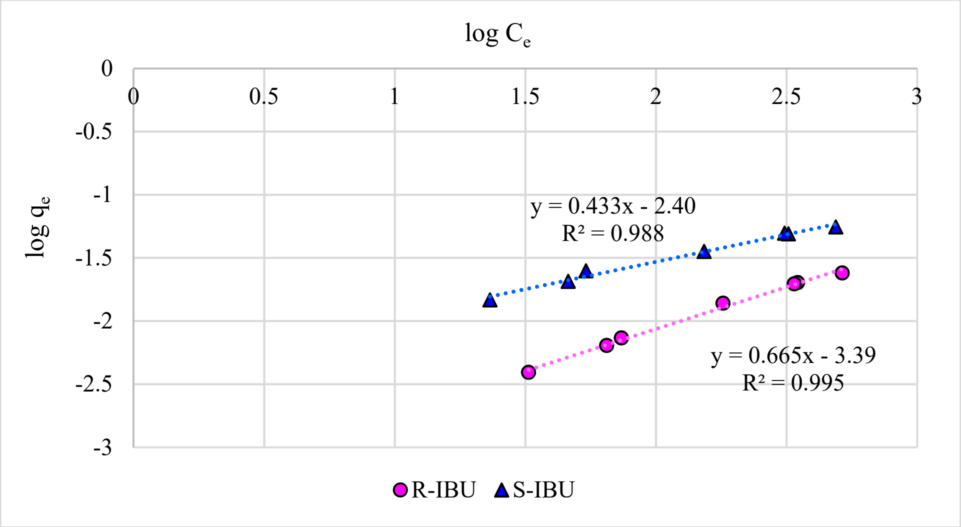

Figure S10. Linearized Freundlich isotherm plot of equipment-IBU-NF270 membrane adsorption relationship.

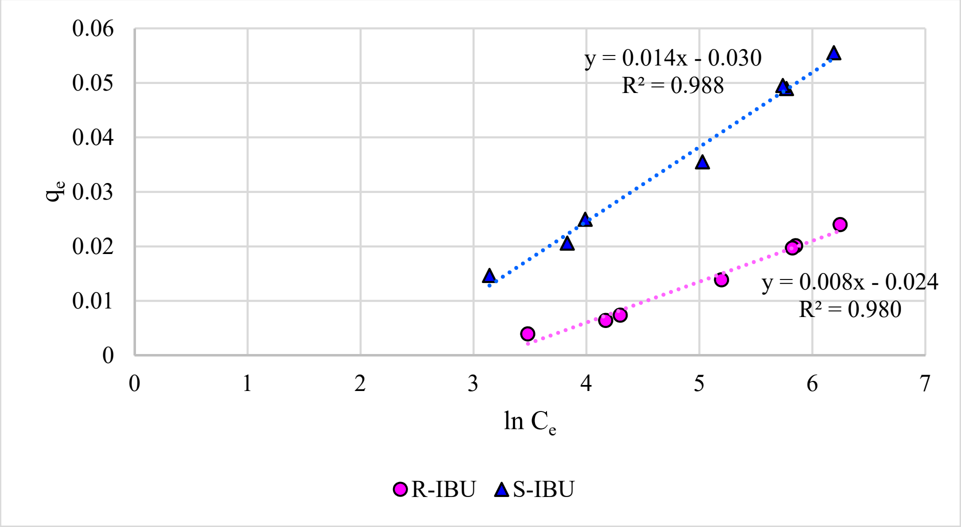

Figure S11. Linearized Temkin isotherm plot of equipment-IBU-NF270 membrane adsorption relationship.

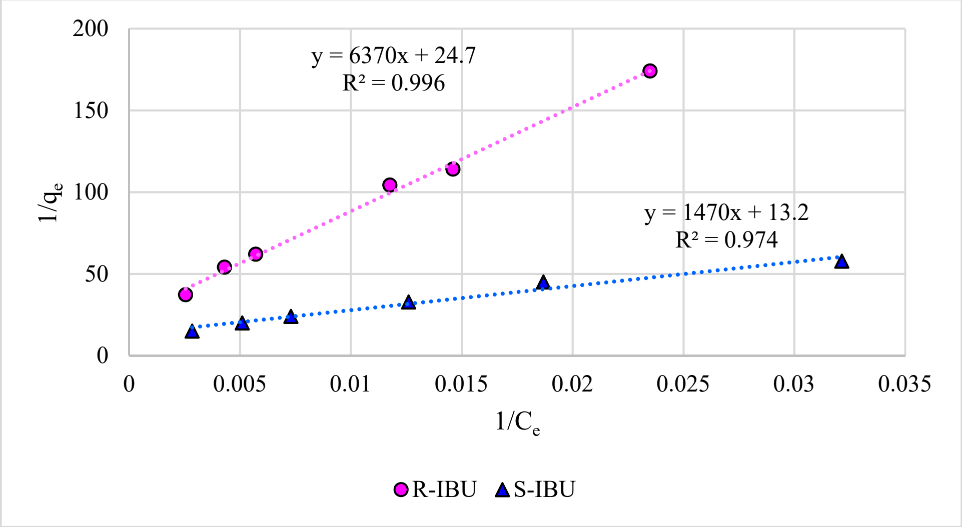

Figure S12. Linearized Langmuir isotherm plot of equipment-IBU-TS40 membrane adsorption relationship.

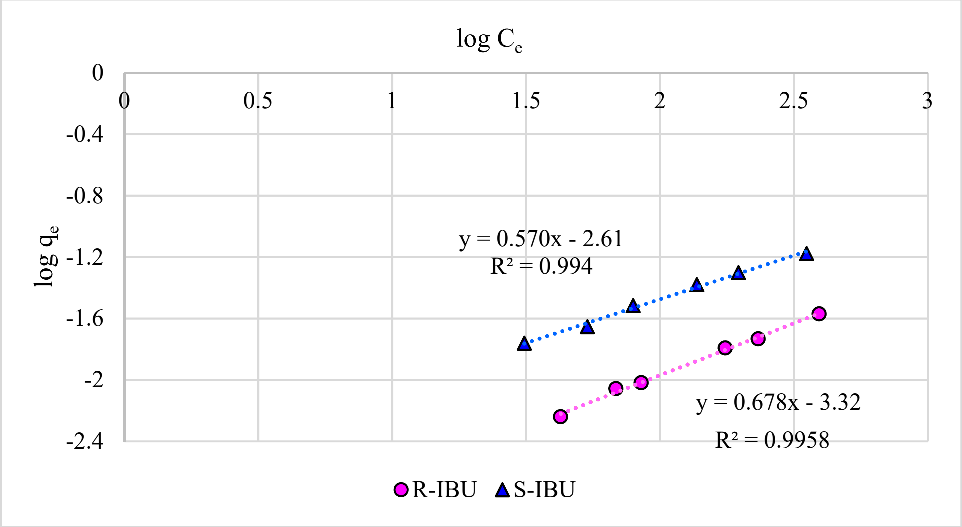

Figure S13. Linearized Freundlich isotherm plot of equipment-IBU-TS40 membrane adsorption relationship.

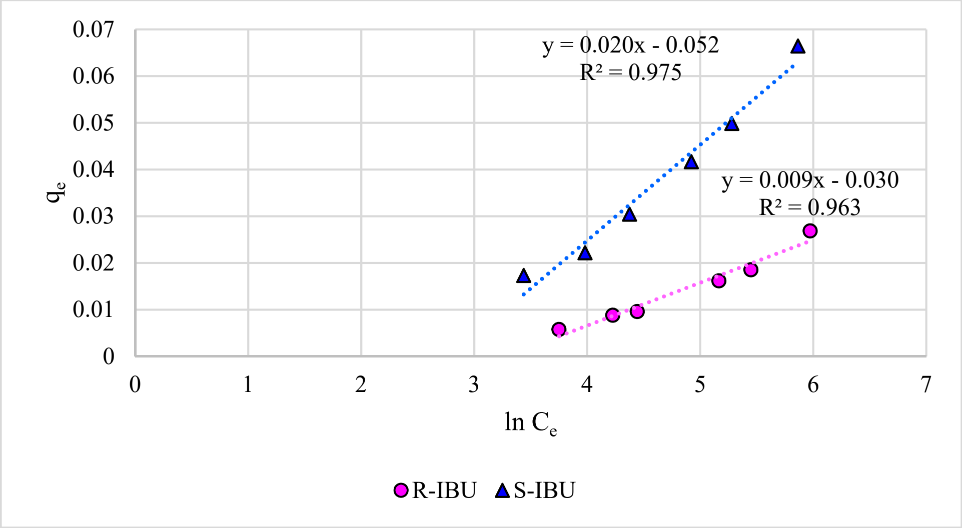

Figure S14. Linearized Temkin isotherm plot of equipment-IBU-TS40 membrane adsorption relationship.

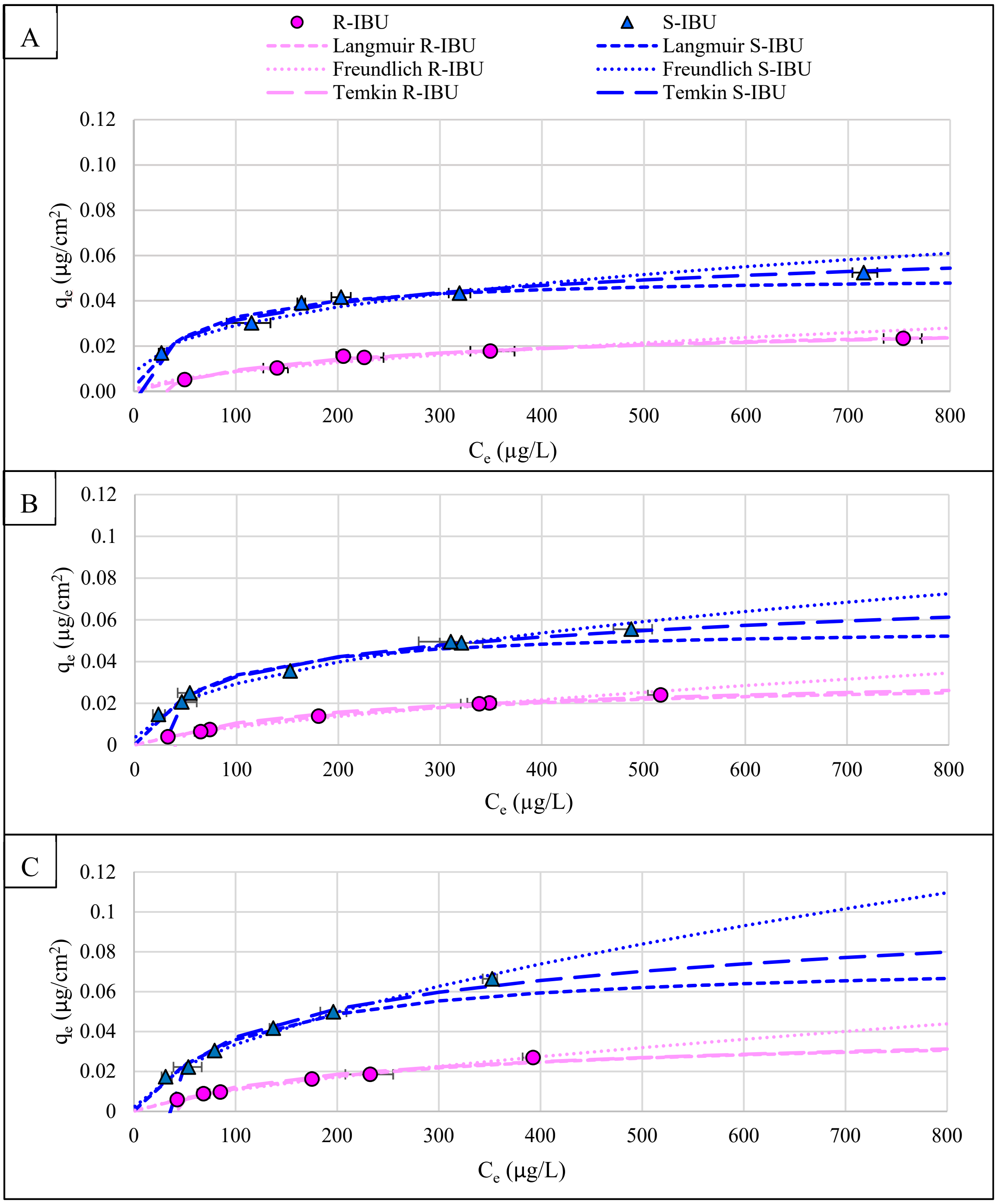

Figure S15. Adsorption isotherm curves of R- and S-IBU. (**A**) Flat-sheet equipment; (**B**) Flat-sheet equipment and NF270 coupon; (**C**) Flat-sheet equipment and TS40 coupon (feed water pH of 4.0 units, temperature 20 ± 1 °C). Error bars represent minimum and maximum values from triplicate analysis.

Determination of Rejection

Rejection (r) describes the ability of a membrane to remove solute from water and is calculated using Equation (S5) [7].

$$r = \frac{C_F - C_P}{C_F} \times 100 \quad \text{(S5)}$$

Equilibrated IBU rejection was calculated by collecting feed and permeate at 24 h. Hence, the overall rejection due to the adsorptive behavior of IBU could be compared to the actual rejection of the solute at quasi-equilibrium.

References.

1. O'Neil, M.J., Ed. The Merck Index, 13th, ed.; Merck & Co: Whitehouse Station, NJ, USA, 2001; pp 3961–4906.
2. Yalkowsky, S.H.; Dannenfelser, R.M. Aquasol database of aqueous solubility. College of Pharmacy, University of Arizona: Tuscon, AZ, USA, 1991.
3. Avdeef, A.; Box, K.J.; Comer, J.E.A.; Hibbert, C.; Tam, K.Y. pH-Metric logP 10. Determination of Liposomal Membrane-Water Partition Coefficients of lonizable Drugs. *Pharm. Res.* **1998**, *15*, 209–215, doi:10.1023/a:1011954332221.
4. Physical and thermodynamic properties of pure chemicals: Data compilation. *Choice Rev. Online* **1990**, *27*, 27, doi:10.5860/choice.27-3319.
5. Shaw, L.R.; Irwin, W.J.; Grattan, T.J.; Conway, B.R. The Effect of Selected Water-Soluble Excipients on the Dissolution of Paracetamol and Ibuprofen. *Drug Dev. Ind. Pharm.* **2008**, *31*, 515–525, doi:10.1080/03639040500215784.
6. United States Environmental Protection Agency EPI-Suite Estimation Programs Interface Suite™ for Microsoft® Windows. 2008, 4.10.
7. Howe, K.J.; Hand, D.W.; Crittenden, J.C.; Trussell, R.R.; Tchobanoglous, G. Principles of Water Treatment. John Wiley & Sons, Inc.: Hoboken, 2012.
8. Kwok, † D.Y.; Gietzelt, § T.; Grundke, § K.; Jacobasch, § A.H.-J.; Neumann, † A.W. Contact Angle Measurements and Contact Angle Interpretation. 1. Contact Angle Measurements by Axisymmetric Drop Shape Analysis and a Goniometer Sessile Drop Technique. *Langmuir* **1997**, *13*, 2880–2894, doi:10.1021/la9608021.
9. Al-Amoudi, A.; Williams, P.; Al-Hobaib, A.; Lovitt, R.W. Cleaning results of new and fouled nanofiltration membrane characterized by contact angle, updated DSPM, flux and salts rejection. *Appl. Surf. Sci.* **2008**, *254*, 3983–3992, doi:10.1016/j.apsusc.2007.12.052.
10. Hashim, N.H.; Khan, S.J. Enantioselective analysis of ibuprofen, ketoprofen and naproxen in wastewater and environmental water samples. *J. Chromatogr. A* **2011**, *1218*, 4746–4754, doi:10.1016/j.chroma.2011.05.046.
11. Kumar, K.V.; Sivanesan, S. Comparison of linear and non-linear method in estimating the sorption isotherm parameters for safranin onto activated carbon. *J. Hazard. Mater.* **2005**, *123*, 288–292, doi:10.1016/j.jhazmat.2005.03.040.
12. Subramanyam, B.; Das, A. Linearised and non-linearised isotherm models optimization analysis by error functions and statistical means. *J. Environ. Heal. Sci. Eng.* **2014**, *12*, 92, doi:10.1186/2052-336x-12-92.
13. Shikuku, V.O.; Kowenje, C.O.; Kengara, F.O. Errors in Parameters Estimation Using Linearized Adsorption Isotherms: Sulfadimethoxine Adsorption onto Kaolinite Clay. *Chem. Sci. Int. J.* **2018**, *23*, 1–6, doi:10.9734/csji/2018/44087.

.
